#  Misdiagnosis of brown tumour caused by primary hyperparathyroidism: a case report with literature review

**DOI:** 10.1186/s12902-022-00971-2

**Published:** 2022-03-14

**Authors:** Yanchun Zhong, Yuxi Huang, Jiaquan Luo, Yongjun Ye

**Affiliations:** 1 Department of spine surgery, First Affiliated Hospital of Gannan Medical University, No. 128 Jin Ling Road, 341000 Ganzhou, Jiangxi China; 2 Department of basic medicine, Gannan Health Vocational College, No. 12 Rong Jiang Road, 341000 Ganzhou, Jiangxi China

**Keywords:** Brown tumour, Primary hyperparathyroidism, Misdiagnosis, Parathyroid carcinoma, Case report

## Abstract

**Background:**

Brown tumour is a rare tumour-like lesion of the bone, which is considered as an end-stage lesion of abnormal bone metabolism caused by persistently high parathyroid hormone (PTH) levels. Brown tumour can be found in any part of the skeleton; in some cases, it can occur in multiple bones and can be easily misdiagnosed as a metastatic tumour.

**Case presentation:**

We report the case of a 44-year-old man who presented to the Department of Oncology in our hospital with a 2-month history of local pain in his left shoulder joint. The initial diagnosis was an aneurysmal bone cyst by biopsy, for which the patient underwent tumour resection surgery. The diagnosis of a malignant tumour was made again following postoperative pathological examination. The pathological sections and all clinical data were sent to the Department of Pathology of the First Affiliated Hospital of Sun Yat-sen University; the diagnosis made there was brown tumour. His blood PTH level was 577 pg/ml (15–65 pg/ml). Colour Doppler ultrasonography of the parathyroid gland suggested a parathyroid adenoma. For further treatment, the left parathyroid adenoma was removed by axillary endoscopic resection. Postoperatively, a pathologic examination was performed, and the diagnosis of a parathyroid adenoma was confirmed. One year after the surgery, the left humerus was completely healed, and the left shoulder joint had a good range of movement.

**Conclusions:**

In summary, histopathological diagnosis is not sufficient for the diagnosis of brown tumours. A comprehensive analysis combining clinical symptoms with findings of imaging and laboratory tests is also required. Generally, the treatment of brown tumour includes only partial or complete resection of the parathyroid glands. However, when the tumour is large, especially when it involves the joint, surgery is indispensable.

## Background

Brown tumour is a rare tumour-like lesion of the bone, which is considered as an end-stage lesion of abnormal bone metabolism caused by persistently high parathyroid hormone (PTH) levels. The incidence of primary hyperparathyroidism (PHPT) is lower (< 5%) than that of secondary hyperparathyroidism [1, 2]. Brown tumour can be found in any part of the skeleton; in some cases, it can be located in multiple bones and is easily misdiagnosed as metastatic tumour [3, 4]. In addition, the symptoms of brown tumour are atypical, characterized by local swelling, pain, and sometimes even pathologic fractures.

Generally, the diagnosis of brown tumour is based on medical history, clinical examination, laboratory results, and imaging findings. Pathological examination is the gold standard for diagnosis, which shows mononuclear cells mixed with multinuclear giant cells [5]. However, these findings are similar to those of giant cell tumours (GCTs) and may lead to a wrong diagnosis [6]. In addition, the imaging findings of brown tumour are similar to those of osteolytic disease, including primarily bone metastasis, amyloid cysts, chondroma, aneurysmal bone cyst, osteosarcoma, and GCT or myeloplax tumours, which appear as limited osteolytic lesions [7, 8]. Moreover, the most important element for the diagnosis of brown tumours is recognising PHPT with extremely high PTH values and serum calcium levels. However, PTH is not routinely checked and can be easily overlooked. Therefore, it is a challenge for surgeons to diagnose brown tumour. Treatment of hyperparathyroidism is the best treatment for brown tumour; in response, brown tumour regresses spontaneously [9, 10].

Herein, we described a case that emphasizes the importance of considering PHPT in the differential diagnosis of patients with multiple lytic bone lesions, thus avoiding unnecessary and harmful interventions.

## Case presentation

**Fig. 1:**
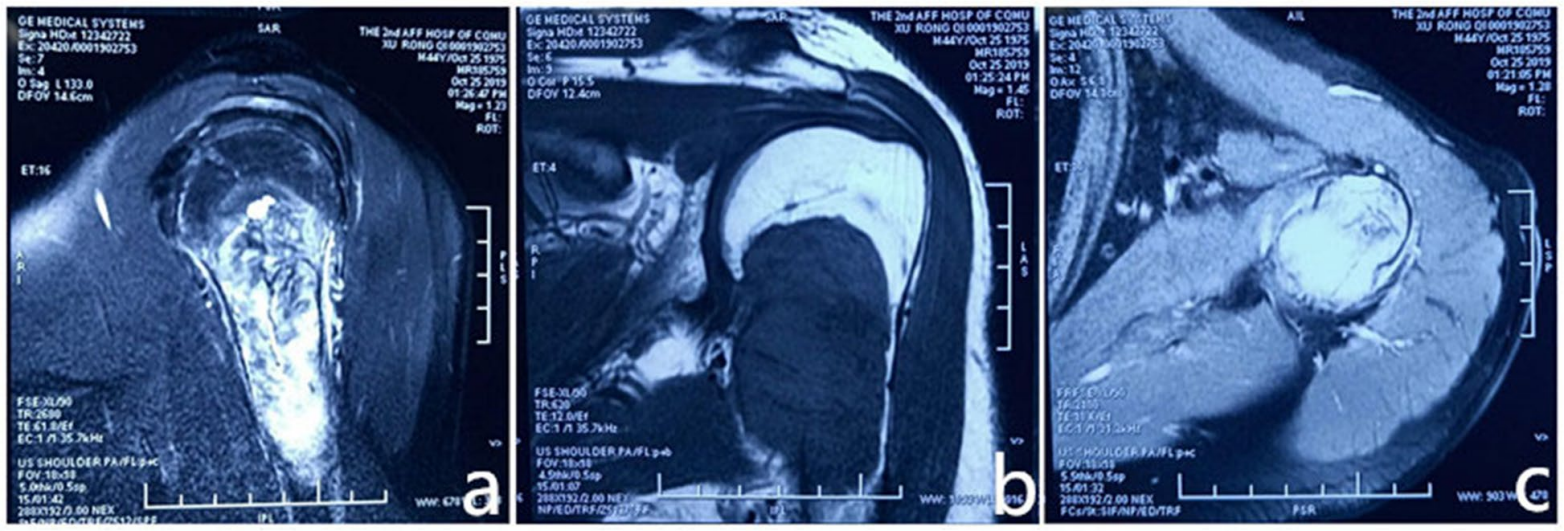
Magnetic resonance imaging (MRI) of the proximal left humerus. **a** Sagittal, **b** coronal, and **c** transversal MRI of the proximal left humerus. MRI shows a 70 × 40 mm mass, with an unclear boundary and mixed hyperintense signals on T2-weighted imaging (T2WI) and hyperintense signals on T1-weighted imaging (T1WI) of the proximal left humerus

A 44-year-old man presented to the Department of Oncology in our hospital with a 2-month history of local pain of the left shoulder joint. He initially visited a local hospital, where magnetic resonance imaging (MRI) was performed (Fig. 1). A 70 × 40 mm mass with an unclear boundary, a mixed hyperintense signal on T2-weighted imaging (T2WI), and a hyperintense signal on T1-weighted imaging (T1WI) of the proximal left humerus was observed. In addition, the adjacent bone was damaged, and a patchy bone marrow oedema signal was observed. This mass presented a state of expansion growth and cortical osteolysis. Based on these results, the patient was diagnosed with a malignant bone tumour by a local doctor. For further treatment, the patient was referred to a superior hospital.

**Fig. 2:**
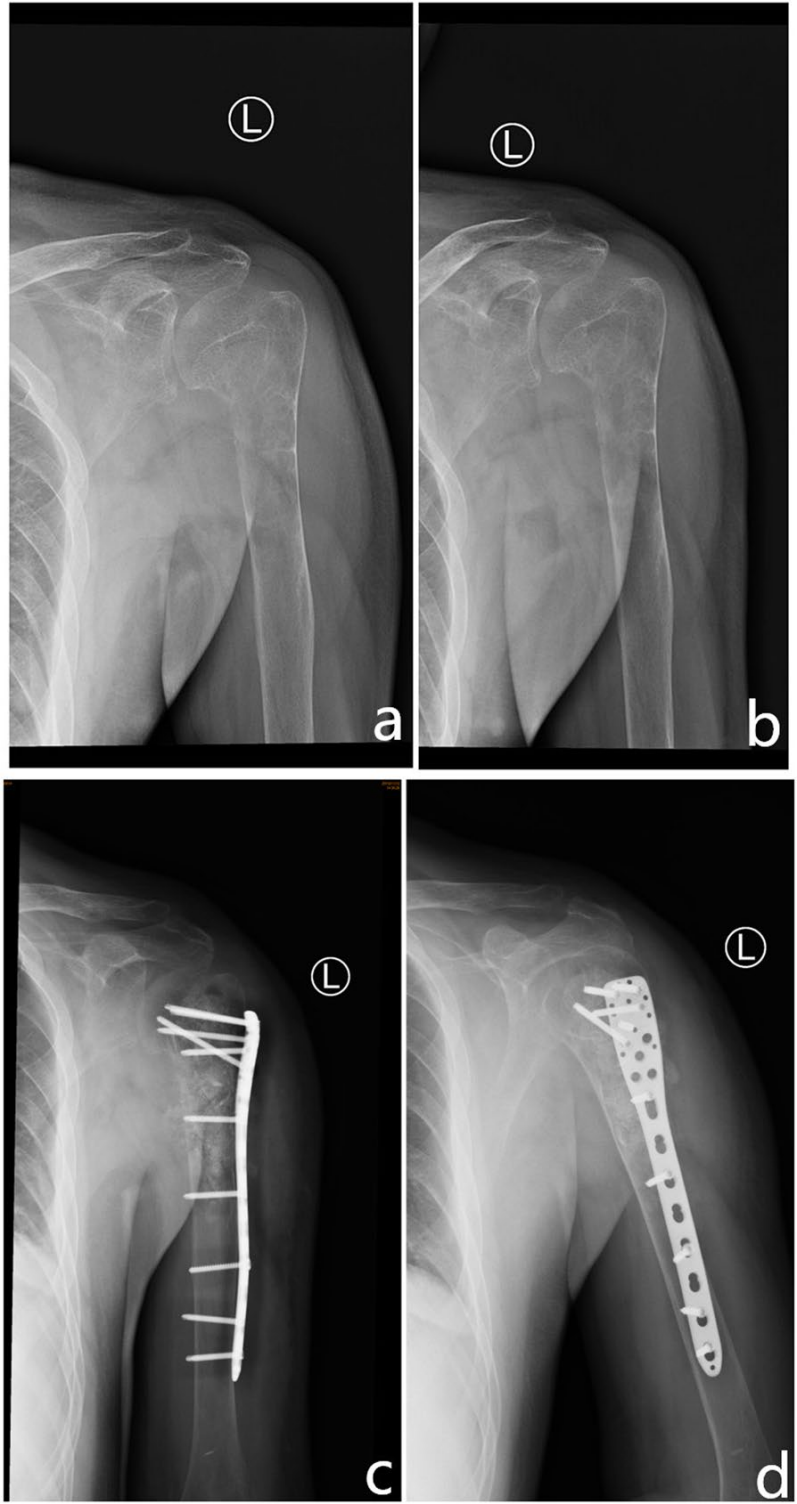
Radiography of the involved bones. Standard **a** anteroposterior and **b** oblique radiographs of the left shoulder joint showing large osteolytic lesions involving the proximal humerus and humeral head, without any joint involvement. **c**, **d** Postoperative radiograph showing resection of the proximal part of the left humerus. Internal fixations were installed to stabilize the bone

**Fig. 3:**
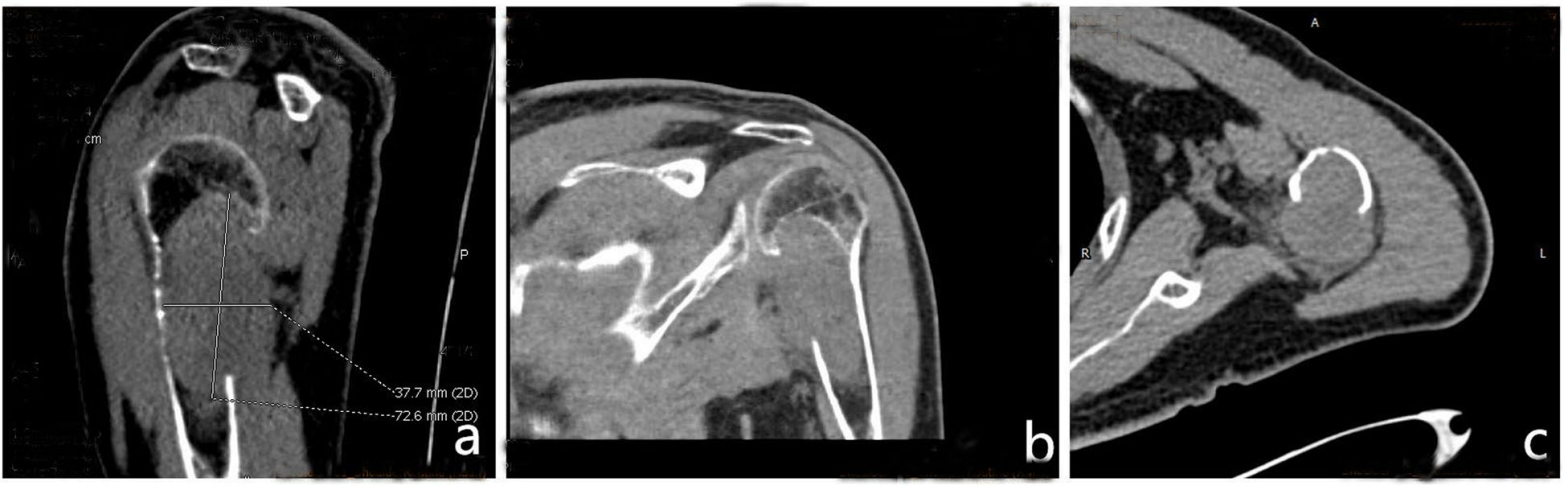
Computed tomography of the left shoulder joint. **a** Sagittal, **b** coronal, and **c** transversal computed tomography (CT) of the left shoulder joint showing a soft tissue mass of approximately 34 × 70 mm in the medullary cavity

**Fig. 4:**
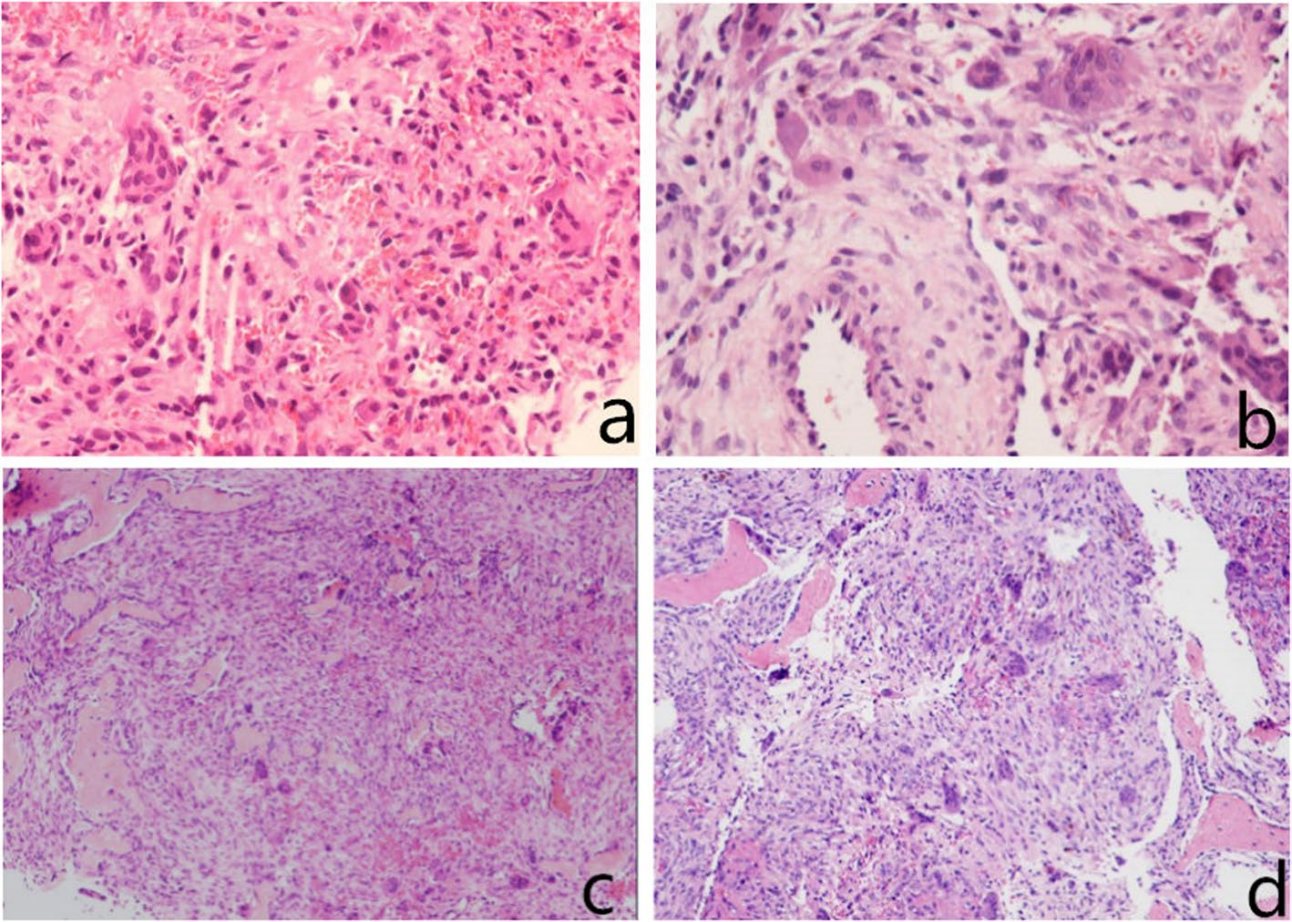
Ultrasonography and pathological findings. **a, b** A colour ultrasound-guided puncture biopsy of the proximal left humerus showing a brown spongy material comprising several multinucleated giant cells, without atypia. **c, d** The diagnosis after the postoperative pathological examination was a malignant fibrous histiocytoma

His physical examination on admission revealed that the left shoulder joint and proximal superior arm were slightly swollen and tender, and the local skin temperature was normal. Shoulder lifting was limited. The patient’s body mass index was 21.4 kg/m^2^. His blood investigations revealed the following: calcaemia, 3.09 mmol/L (2.11–2.52 mmol/L); phosphoraemia, 0.55 mmol/L (0.85–1.51 mmol/L); and alkaline phosphatase level, 461 U/L (45–125 U/L). The other investigation results were normal. Standard anteroposterior and oblique radiographs of the left shoulder joint showed large osteolytic lesions involving the proximal humerus and humeral head without any joint involvement (Fig. 2a, b). Subsequent computed tomography (CT) of the left shoulder joint showed a soft tissue mass of approximately 34 × 70 mm in the medullary cavity (Fig. 3). These imaging findings were suggestive of a bone malignancy. For further diagnosis, a colour ultrasound-guided puncture biopsy of the proximal left humerus was performed, and a brown spongy material consisting of several multinucleated giant cells without atypia was observed. This was suggestive of an aneurysmal bone cyst (Fig. 4a, b).

**Fig. 5:**
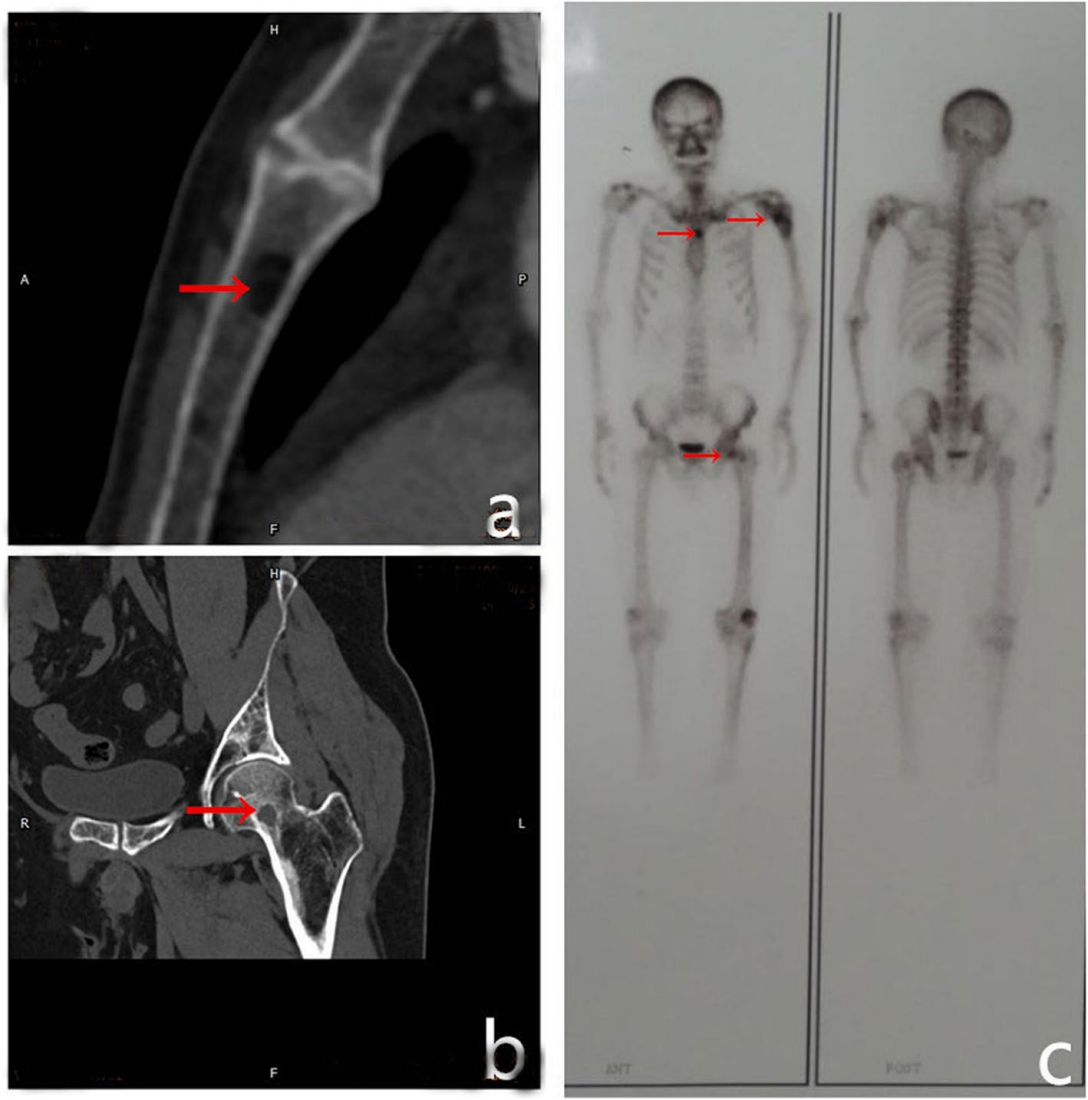
Computed tomography and systemic radionuclide bone scanning of involved bones. **a, b** Computed tomographic scan revealing multiple osteolytic lesions in the patient’s sternum and left femoral neck. **c** Systemic radionuclide bone scanning showing active metabolism of the superior part of the left humerus, sternum, and left femoral neck

After discussion about the choice of treatment, tumour resection with postoperative pathological examination of the lesions was recommended. Therefore, the patient underwent surgery. Intraoperatively, we noticed that the cortical bone of the proximal humerus was thin and brittle. In addition, several cystic cavities filled with brown viscous substances were observed in the medullary cavity, showing honeycomb changes. We excised all the lesions, and an autologous bone was implanted in the cavity. Subsequently, proper internal fixations were installed to stabilise the bone (Fig. 2c, d). A postoperative pathological examination was performed again, and the findings were suggestive of a malignant fibrous histiocytoma of the left humerus (Fig. 4c, d). Systemic radionuclide bone scanning was performed to further exclude other bone diseases as the pre- and postoperative diagnoses differed. It showed active metabolism of the superior part of the left humerus, sternum, and left femoral neck (Fig. 5c). CT revealed multiple osteolytic lesions in the sternum and left femoral neck (Fig. 5a, b).

**Fig. 6:**
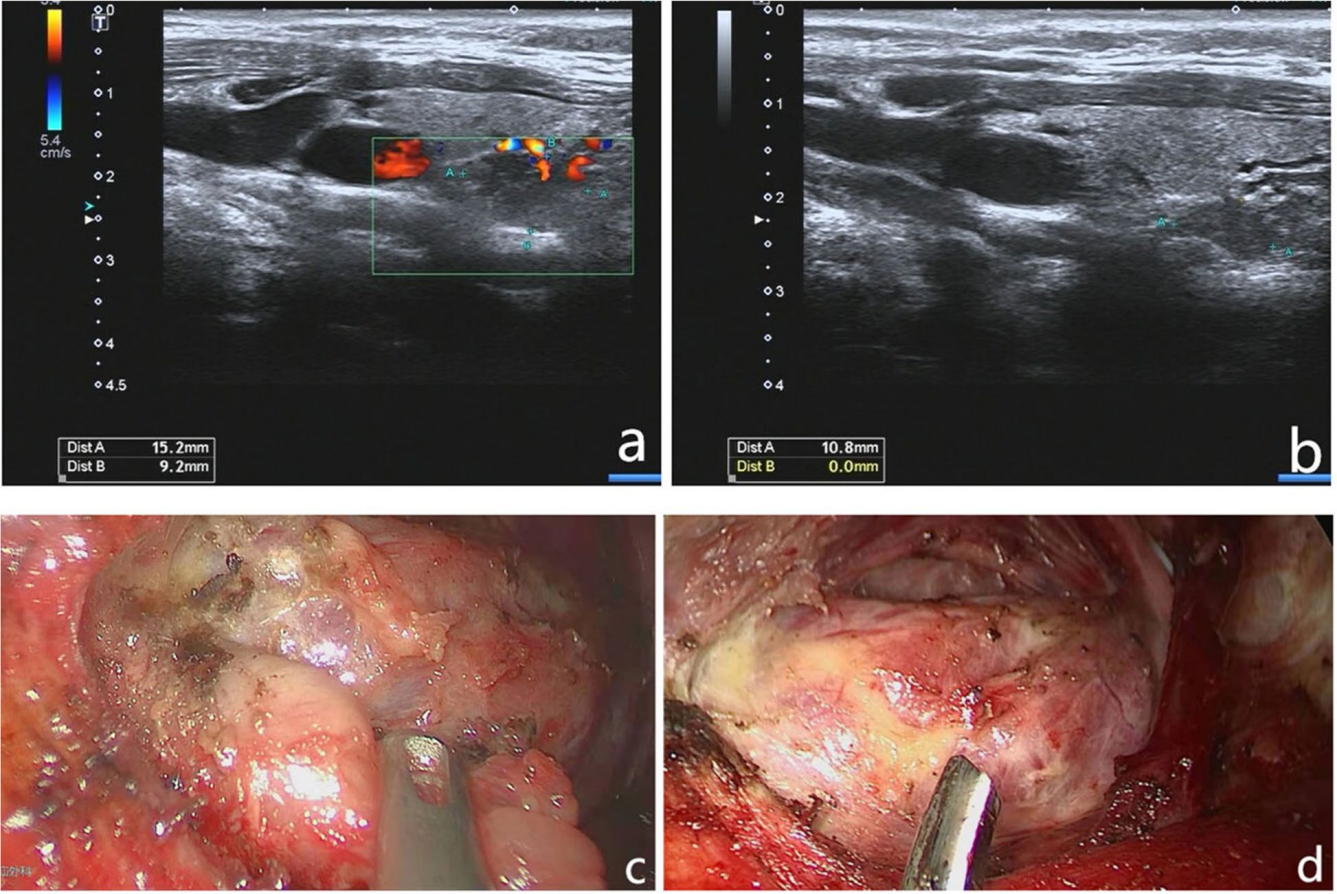
Colour doppler ultrasonography of the parathyroid gland and thyroid gland. **a, b** Colour Doppler ultrasound examination of the parathyroid gland showing that the left dorsal thyroid is hypoechogenic, with a size of 17 × 12 mm, an unclear boundary, and irregular shape. This was diagnosed as a parathyroid adenoma. **c, d** Solid yellow nodules with clear boundaries are observed at the left dorsal lobe of the thyroid gland

Due to the limited diagnostic level in our hospital, we sent the pathological sections and clinical data to the Department of Pathology of the First Affiliated Hospital of Sun Yat-sen University; the suggested diagnosis was brown tumour. They suggested we perform further parathyroid function tests. His blood PTH level was 577 pg/ml (15–65 pg/ml). Colour Doppler ultrasonography of the parathyroid gland showed that the left dorsal thyroid was hypoechogenic, 17 × 12 mm, with unclear boundaries and an irregular shape, which was considered as a parathyroid adenoma (Fig. 6a, b). Ultimately, the confirmed diagnosis was a brown tumour caused by PHPT.

**Fig. 7:**
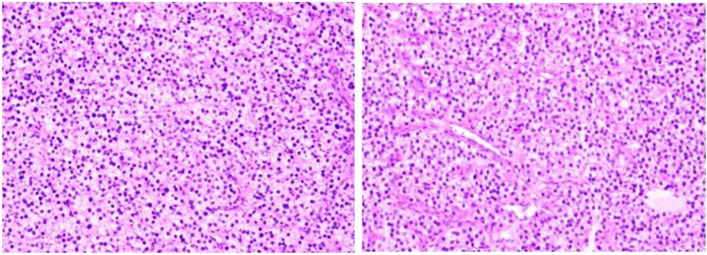
Pathologic examination of the solid yellow nodules

Regarding the treatment of PHPT, the patient was transferred to the Department of Otolaryngology for surgery. The left parathyroid adenoma was removed by axillary endoscopic resection. Intraoperatively, 20 × 10 mm solid yellow nodules with clear boundaries were observed at the left dorsal lobe of the thyroid gland (Fig. 6c, d). The parathyroid adenoma was completely removed after separation. A postoperative pathologic examination was conducted, which proved to be a parathyroid adenoma (Fig. 7). The PTH levels dropped to 29 pg/ml (15–65 pg/ml) 4 days after surgery.

**Fig. 8:**
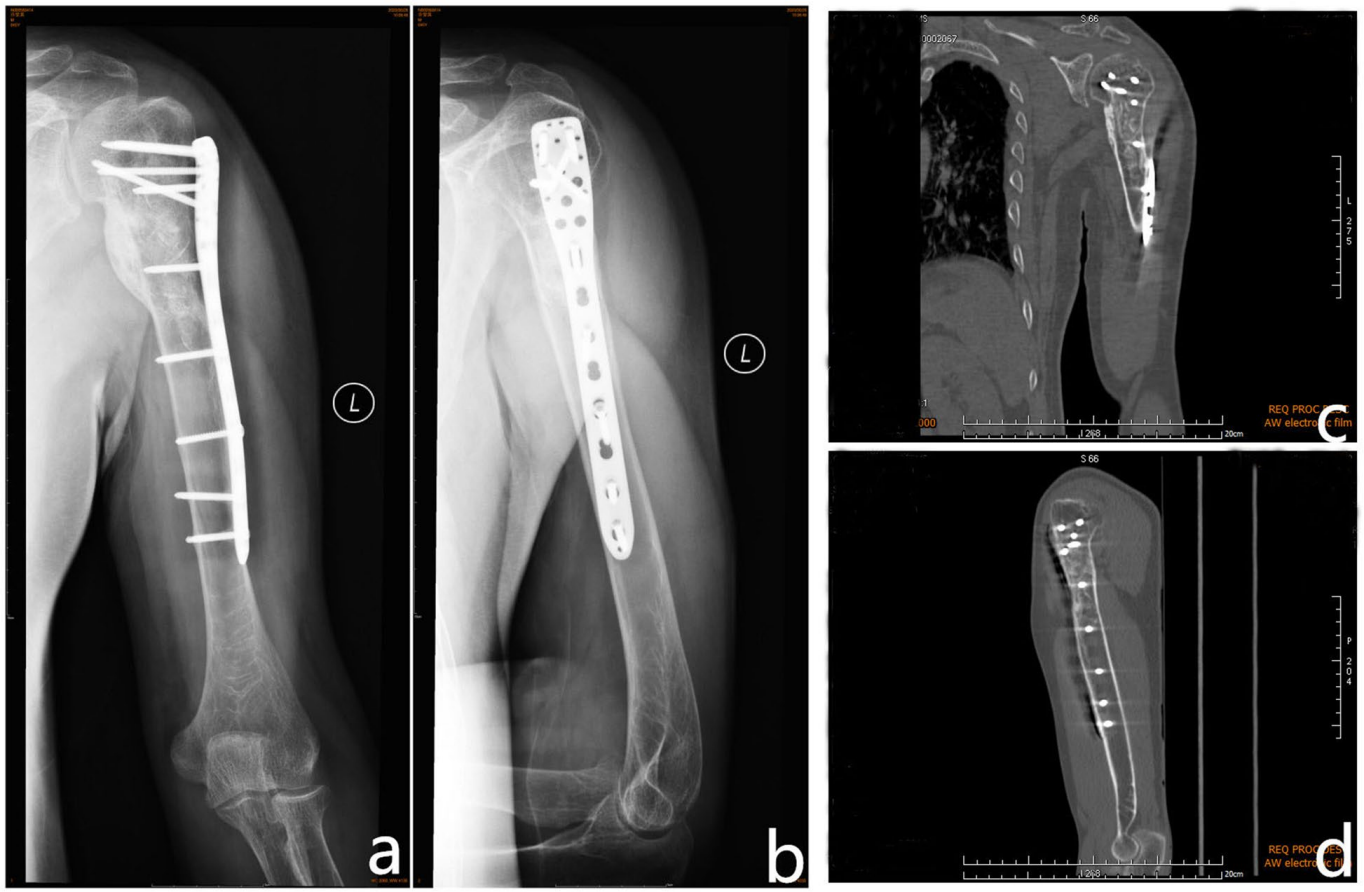
Radiography and computed tomography findings of brown tumour in the left humerus. One year after the operation, radiography **a, b** and computed tomographic scan **c, d** show regression of the brown tumour and no new bone lesions in the left humerus

One year after the surgery (Fig. 8), the left humerus lesions had healed completely, and the left shoulder joint had a good range of movement.

## Discussion and Conclusions

PHPT is the third most common endocrine disease after diabetes and thyroid disease. Its incidence ranges from 0.025 to 0.065%, with the highest incidence in postmenopausal women [11–13]. The abnormally increased secretion of PTH usually affects calcium levels, phosphate levels, and bone metabolism. It usually causes renal and skeletal manifestations. However, due to the routine blood calcium and phosphorus tests, PHPT is generally detected in the early stage. Therefore, most patients with PHPT are asymptomatic [14].

Brown tumour, which is one of the pathognomonic signs of PHPT, is a focal, tumour-like bony lesion caused by osteoclastic turnover of bone. Although it is reported that musculoskeletal manifestations in patients with PHPT can reach 54.7%, the incidence rate of brown tumour ranges from 1.5 to 4.5% [15, 16]. In addition, the symptoms of brown tumour are atypical, characterized by local swelling, pain, and sometimes even pathologic fractures. Thus, the misdiagnosis rate increases when the surgeon is inexperienced in diagnosing brown tumours.

To increase the diagnostic rate of brown tumours, some diagnostic methods are needed in clinical practice, including clinical examination, laboratory investigations, imaging examinations, and pathological examination [17]. Among these methods, pathological examination is the gold standard for the diagnosis of brown tumours [18]. Several cases have been reported by clinicians who initially misdiagnosed brown tumours as other bone diseases; however, these were ultimately proven to be brown tumours by pathological examination [19, 20]. However, in some cases, bone biopsy may be less informative [21–23]. Panagopoulos et al. [21] reported a 53-year-old man who presented to the hospital with a 2-month history of a painful and moderately swollen left wrist. The result of bone biopsy showed a GCT, although it was ultimately diagnosed as a brown tumour by electrolyte examination and elevated levels of PTH. In another report, Schnyder et al. [22] reported a 72-year-old woman who presented with malaise, weight loss, and hypercalcemia, with a history of breast cancer 7 years prior. Preoperative and postoperative bone biopsies did not suggest either malignancy or brown tumour. Healing of the bone lesions after neck surgery eventually supported the diagnosis of brown tumour. Lastly, Bohdanowicz-Pawlak et al. [23] reported a patient with numerous osteolytic lesions in the skull bones and other multiple sites. It was difficult to differentiate the lesions from other giant cell lesions using pathologic findings. Eventually, electrolyte investigation results were suggestive of brown tumour. In the meantime, when faced with a patient with end-stage renal disease, physicians should include brown tumour as a differential diagnosis [24]. We found that brown tumours and other bone diseases such as GCT and metastatic tumours share similar pathologic findings, and differential diagnosis can be extremely difficult. Similar to this patient, the diagnosis was made based on pathological findings alone. However, the two diagnoses made on pathological examination were aneurysmal bone cyst and malignant fibrous histiocytoma, respectively, which were incorrect. Although the radiological findings, including 99mTc-MIBI SPECT/CT scintigraphy, and clinical data were not specific, they could be useful as adjunct tests for brown tumours [7, 8, 25, 26]. Therefore, radiological findings and clinical data are important in guiding the pathologist when the diagnosis is unclear. In addition, serum calcium and PTH measurements are also important diagnostic tools [27–29].

Controversies on the use of surgery and conservative treatment for brown tumour still exist. Generally, brown tumour does not need surgical treatment because it regresses spontaneously after partial or complete resection of the parathyroid glands [30]. Hu et al. [31] reported a 50-year-old woman who complained of left elbow and thoracodorsal pain with bilateral lower limb weakness, who was admitted to the hospital and diagnosed with multifocal brown tumour. She received intramuscular injection of Miacalcic and incense of Calcitonin (Salmon) Nasal Spray to decrease serum calcium levels. Surgery was performed later to excise the ectopic parathyroidoma. At her 1-year follow-up, the bone lesions disappeared completely. Irie et al. [32] reported a 31-year-old woman who complained of right gonalgia without any trauma history. She was diagnosed with brown tumour based on the imaging and laboratory findings. One month after parathyroidectomy, the gonalgia had resolved completely. Therefore, the key treatment of brown tumour is surgical removal of the hyperfunctioning parathyroid gland [33]. Treatment of hyperparathyroidism is the best treatment for brown tumour; in response, brown tumour regresses spontaneously [9, 10]. However, some patients with large lesions still need surgical interventions, including tumour curettage, Palacos plombage, and less invasive stabilisation systems [34, 35]. In this patient, the lesions of the proximal left humerus were resected. Although the patient was initially misdiagnosed with other bone diseases, surgery was also suitable for brown tumour in our case because the bone lesions were very large and could easily cause pathologic fractures. Panagopoulos et al. [5] reported a 53-year-old man, who was initially misdiagnosed with GCT and underwent unnecessary and harmful surgical interventions. Therefore, a high index of suspicion is required for diagnosing brown tumour at the early stages.

In summary, we described a rare case of brown tumour caused by PHPT, which was initially misdiagnosed as other bone diseases. Clinically, histopathological diagnosis is not enough for the diagnosis of brown tumours. A comprehensive analysis combining clinical symptoms, imaging, and laboratory tests is also required. Particularly, the possibility of bone metabolic diseases, especially brown tumour caused by hyperparathyroidism, should be considered when multiple osteolytic bone destruction occurs throughout the body, with extensive osteoporosis, hypercalcaemia, hypokalaemia, and high alkaline phosphatase levels. Generally, the treatment of brown tumour includes partial or complete resection of the parathyroid glands alone. However, when the tumour is large, especially when it involves the joint, surgery is indispensable.

## Data Availability

Data sharing is not applicable to this article as no datasets were generated or analysed during the current study.

## Abbreviations

PTH: Parathyroid hormone
PHPT: Primary hyperparathyroidism
GCTs: Giant cell tumours
MRI: Magnetic resonance imaging
T2WI: T2-weighted imaging
T1WI: T1-weighted imaging
CT: Computed tomography
